# A Novel Data-Driven Algorithm for Prediction Horizon Estimation in Model Predictive Control

**DOI:** 10.3390/s26134214

**Published:** 2026-07-03

**Authors:** Bojan Jorgovanović, Nikola Jorgovanović, Darko Stanišić, Luka Mejić

**Affiliations:** 1 Faculty of Technical Sciences, University of Novi Sad, 21000 Novi Sad, Serbia; nikolaj@uns.ac.rs (N.J.); mejic@uns.ac.rs (L.M.); 2 Global Electronic Solutions doo, 21000 Novi Sad, Serbia; darko.stanisic@globalelectronic.rs

**Keywords:** model predictive control, long short-term memory, prediction horizon estimation, data-driven modelling, cross-correlation analysis, data-driven tuning, closed-loop control

## Abstract

**Highlights:**

**What are the main findings?**

- A novel algorithm for prediction horizon estimation in MPC is proposed, based on cross-correlation analysis of data simulated by a trained LSTM model, requiring no experiments on the physical system.
- The algorithm is validated on two nonlinear industrial benchmark systems (CSTR and single tank), with closed-loop MPC simulations confirming strong control performance with the estimated prediction horizons.

**What are the implications of the main findings?**

- LSTM-based process models can be leveraged not only for control but also for systematic MPC tuning, offering a practical and fully data-driven alternative to manual or heuristic parameter selection.
- The offline nature of the method allows the prediction horizon to be determined systematically prior to deployment, rather than through iterative trial-and-error adjustment.

**Abstract:**

Model predictive control (MPC) is a widely used advanced control strategy in industrial applications. The prediction horizon is one of its most influential tuning parameters, as it directly affects both control performance and computational demand. Despite its importance, systematic methods for its configuration remain scarce in the literature. This paper proposes a novel algorithm for prediction horizon estimation based on cross-correlation analysis of input and output data simulated by a trained long short-term memory (LSTM) network model of the controlled process. The use of LSTM networks allows the method to simulate process behaviour directly, eliminating the need for experiments on the physical system. Furthermore, this enables the method to work entirely offline, allowing the prediction horizon to be determined prior to deployment. The proposed algorithm is evaluated on two representative benchmark systems: a continuous stirred-tank reactor and a single tank system. LSTM models are trained for both benchmark systems and are subsequently integrated into an MPC framework. Closed-loop simulations demonstrate that MPC controllers designed with the estimated prediction horizons achieve strong control performance across both benchmark systems. The results suggest that cross-correlation analysis of LSTM-simulated data provides a reliable and systematic basis for prediction horizon estimation, contributing a practical tool for MPC tuning in industrial process control.

## 1. Introduction

Model predictive control (MPC) is an advanced control technique that determines the optimal input sequence by solving an optimization problem based on a predictive model of the controlled system [1]. At each sampling instant, the controller determines an optimal sequence of control inputs that minimizes a specified cost function while respecting system constraints. A defining feature of MPC is the use of the receding horizon principle. At each time step, the optimization problem is solved over a finite future time window, and only the first element of the optimized control sequence is applied to the system. At the next sampling instant, the horizon is shifted forward, and the optimization is repeated using updated measurements.

In classical MPC formulations, models are typically derived from first principles, resulting in linear or nonlinear state-space representations based on physical laws. While such models describe the system dynamics using physical relationships, their development can be challenging for complex and highly nonlinear processes [2]. As an alternative, data-driven modelling approaches have gained significant attention in MPC [3,4,5]. These methods rely on measured input and output data to construct predictive models without requiring explicit knowledge of the underlying governing equations. Among data-driven techniques, artificial neural networks have proven particularly effective due to their universal approximation capability and flexibility in capturing nonlinear dynamics. Feedforward neural networks (FFNNs) have been widely used for both modelling industrial processes [6,7,8] and providing real-time estimations as soft sensors [9,10]. However, as pointed out in [2], their ability to represent temporal dependencies is limited when applied to dynamic systems. In contrast, recurrent neural networks (RNNs) provide a natural framework for modelling dynamical systems due to their internal memory structure. As a result, they have been extensively used for dynamic system identification and nonlinear process modelling. For instance, in [11], the authors developed a simple RNN-based model of a fixed-bed reactor and used it in an MPC framework, achieving a robust and effective process control approach. Similar results were presented in [12], where the authors modelled a continuous stirred-tank reactor for pharmaceutical manufacturing. In [13] the authors presented a new way of utilizing simple RNNs by slightly altering the architecture by incorporating a priori knowledge about the modelled process. In [14], the authors presented an MPC framework which utilizes simple RNNs for modelling batch crystallization processes. Even though they are widely used for modelling, simple RNNs suffer from the vanishing and exploding gradient problem, which can hinder their ability to learn long-term temporal dependencies. Long short-term memory (LSTM) networks are specifically designed to capture long-term temporal dependencies and mitigate the mentioned issues, making them well suited for systems with delays, slow dynamics, or strong temporal correlations [15]. As a result, LSTM-based models have been increasingly adopted as prediction models within MPC frameworks, enabling accurate multi-step predictions directly from historical data [3]. In [16], the authors provide a stability analysis of LSTM networks when used as process models in MPC and give an example with a pH neutralization process. In [17], LSTM networks were used to model a reverse osmosis desalination plant which was used for MPC to successfully track the reference signal. In [18], the authors developed an algorithm for training LSTM networks with noisy data, thereby teaching the networks to predict the underlying process dynamics. The algorithm’s effectiveness was tested by performing MPC on a chemical reactor process. Other than industrial processes, LSTMs have also been used for MPC of heating, ventilation and air conditioning (HVAC) systems, where they have shown significant reductions in energy consumption [19,20,21].

In addition to the choice of the prediction model, the performance of an MPC controller strongly depends on the selection of a suitable prediction horizon. The prediction horizon determines how far into the future the system behaviour is predicted and optimized, and therefore directly influences the controller’s ability to anticipate system dynamics, constraints, and disturbances. An inadequately short prediction horizon may prevent the controller from accounting for slow dynamics, transport delays, or long-term effects of control actions, leading to suboptimal or even unstable closed-loop behaviour. Conversely, an excessively long prediction horizon can significantly increase computational complexity without necessarily providing proportional improvements in control performance. From a control perspective, the horizon should be sufficiently long to capture the dominant dynamics of the system and allow the controller to foresee the impact of current control actions on future outputs [22,23]. This is particularly important for systems with transport delays, integrating behaviour, or strong nonlinearities, where the effect of an input may only become apparent after several sampling intervals. From a computational standpoint, increasing the prediction horizon enlarges the underlying optimization problem, which may limit real-time applicability, especially for nonlinear MPC formulations. Despite being an important parameter, its selection is often based on heuristic rules [24], empirical tuning [25], or simply prior experience. In many cases, determining a suitable prediction horizon requires experimental analysis of the system, where sufficiently exciting input signals are applied in order to reveal the relevant dynamic behaviour. However, such experiments may be costly, time-consuming, or even infeasible in practice, particularly for industrial processes operating under strict constraints. As a result, the prediction horizon is frequently chosen in an ad hoc manner, without a systematic guarantee that it provides an appropriate balance between control performance and computational complexity. In recent years, most methods found in the literature revolve around dynamically adjusting the prediction horizon in order to optimize the MPC performance rather than setting it beforehand. In [26,27], the authors showed how reinforcement learning and proximal policy optimization, respectively, can be used for these adjustments. In these situations, the optimal prediction horizon is learned and depends on the state of the controlled system. Similarly, Ref. [28] utilized an FFNN to dynamically set the prediction horizon based on the system state. Alternatively, in [29,30,31], the authors used different optimization methods to determine optimal prediction horizons in their respective control problems. In [32], the authors used the autoregressive model with exogenous inputs of a building’s thermal inertia to dynamically update the prediction horizon for MPC in HVAC cases. A different approach is proposed in [33,34,35], where Laguerre functions are used to parameterise the degrees of freedom in MPC, implicitly embedding an infinite prediction horizon through the Laguerre basis without requiring the user to specify it explicitly.

While the mentioned approaches demonstrate promising results, they also exhibit certain limitations that may restrict their applicability in practical process industry settings. Methods based on reinforcement learning inherently require the system to be excited and driven through a wide range of operating regimes in order to explore the state space and converge to an optimal prediction horizon policy. Similarly, training an FFNN to dynamically adjust the prediction horizon requires the collection of a sufficiently rich and representative dataset, which can only be generated by deliberately perturbing the system across diverse conditions. In process industry applications, such exploratory excitation is not permissible, as industrial systems must operate continuously and safely within tightly constrained bounds. Furthermore, several of the reviewed methods still rely on an arbitrarily chosen initial prediction horizon from which the adaptation procedure is initialized. In an industrial context, even temporarily operating under a poorly chosen prediction horizon can lead to degraded control performance or constraint violations before the method converges to a suitable value. Therefore, the objective of this paper is to propose a new, practical method for estimating an appropriate prediction horizon for MPC applied to industrial processes characterized by significant transport delays and pronounced nonlinearities. The proposed approach determines the prediction horizon directly from the available process model utilizing cross-correlation, which allows the estimation procedure to be performed offline, without requiring additional experiments on the physical process. Furthermore, the proposed method automatically determines a suitable prediction horizon, without requiring subsequent tuning or manual adjustment.

The paper is structured as follows. In Section 2, the methodology of the work is presented, starting with the description of the two benchmark systems used, the generation of data through open-loop simulations and fitting LSTM networks to model the two systems. Then, the cross-correlation based algorithm for prediction horizon estimation is presented along with the evaluation procedure. The results are then presented in Section 3, and the paper is concluded in Section 4.

## 2. Method

This section presents the methodology used to develop and evaluate the proposed prediction horizon estimation algorithm. Firstly, two dynamic nonlinear benchmark systems and their descriptions and associated assumptions are introduced. Secondly, the data generation and model training and testing procedures used to obtain representative system input–output behaviour are described. Thirdly, the proposed prediction horizon estimation algorithm is described in detail. Finally, the algorithm is evaluated by running MPC test case simulations with the trained models and the estimated prediction horizons.

### 2.1. System Descriptions and Assumptions

To evaluate the proposed algorithm in a controlled and reproducible manner, two nonlinear dynamic systems commonly used in MPC-related research are considered. The selected benchmark processes exhibit strong nonlinear behaviour and include non-manipulated inputs. Additionally, artificial transport delays were introduced at each system’s inputs to enable the analysis of the algorithm’s performance on systems with significant time delays. Although transport delays of this nature are commonly encountered in practical industrial applications, the delay values used in this study were selected arbitrarily for benchmarking purposes and do not necessarily correspond to specific physical mechanisms. The transport delays introduced in the benchmark systems were selected such that their magnitudes are of a similar order as the characteristic dynamics of the respective processes. Because of this, neither the transport delay nor the dynamic lag dominate the overall system behaviour. All these characteristics make the benchmark systems suitable for assessing controller performance under realistic operating conditions.

#### 2.1.1. Continuous Stirred-Tank Reactor

The continuous stirred-tank reactor (CSTR) considered in this work is non-isothermal and well mixed. It represents a nonlinear dynamic system which is commonly used as a benchmark in process control research [2]. The reactor performs a reversible first-order exothermic reaction, and its dynamics are described by coupled nonlinear mass and energy balance Equations (1)–(5), discretized in time.

(1) $$C_{A}(k+1)=C_{A}\left(k\right)+T_{p}\left(\frac{1}{\tau }\left(C_{A0}\left(k-200\right)-C_{A}(k)\right)-r_{A}(k)+r_{B}(k)\right)$$

(2) $$C_{B}(k+1)=C_{B}\left(k\right)+T_{p}\left(\frac{-1}{\tau }C_{B}(k)+r_{A}(k)-r_{B}(k)\right)$$

(3) $$T(k+1)=T\left(k\right)+T_{p}\left(\frac{1}{\tau }\left(T_{0}-T\left(k\right)\right)+\frac{-∆H}{\rho C_{p}}\left(r_{A}\left(k\right)-r_{B}(k)\right)+\frac{Q(k-400)}{\rho C_{p}v}\right)$$

(4) $$r_{A}\left(k\right)=k_{A}e^{\frac{-E_{A}}{RT\left(k\right)}}C_{A}(k)$$

(5) $$r_{B}\left(k\right)=k_{B}e^{\frac{-E_{B}}{RT\left(k\right)}}C_{B}(k)$$

The reactor temperature *T* is selected as the controlled variable, while the heating rate *Q* serves as the manipulated variable. The inlet concentration *C_A_*_0_ is considered a non-manipulated external input with unknown dynamics. Transport delays are introduced to both input signals, a delay of 400 sampling instants is applied to the heating rate, and a delay of 200 sampling instants is applied to the inlet concentration. The system is simulated in discrete time with a sampling period of 0.1 s. All system parameter values are summarized in Table 1, and the input ranges are presented in Table 2.

#### 2.1.2. Single Tank System

**Figure 1:**
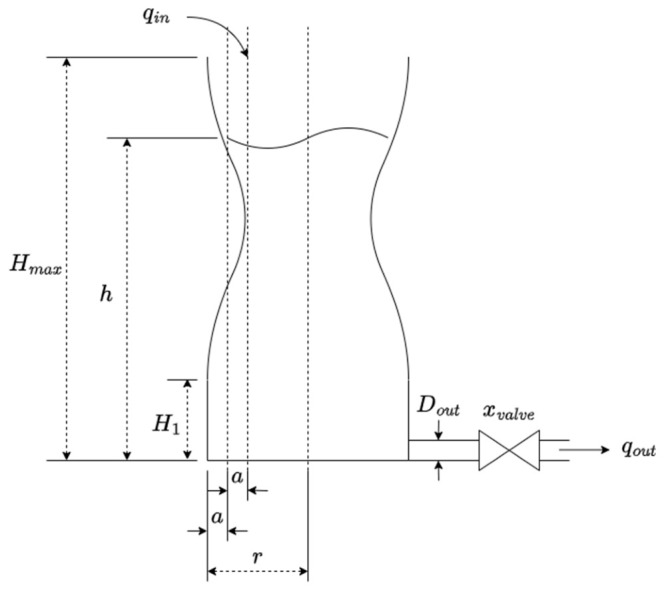
Schematic representation of the used single tank system.

The second benchmark process used in this study is a single tank system based on a standard tank with modifications which include a nonlinear relationship between the liquid level and the tank cross-sectional area, as well as a nonlinear outflow valve characteristic. The system dynamics are described by time-discretized nonlinear Equations (6)–(8) derived from mass balance principles. A diagram of the system is presented in Figure 1.

(6) $$h\left(k+1\right)=h\left(k\right)+T_{p}\left(\frac{\left(q_{in}(k-1000)-q_{out}(k)\right)}{A(h(k))}\right)$$

(7) $$q_{out}\left(k\right)=\sqrt{\frac{x_{valve}(k-500)}{100\%}}\cdot C_{d}{\left(\frac{D_{out}}{2}\right)}^{2}\pi \sqrt{2gh(k)}$$

(8) $$A\left(h(k)\right)=\{\begin{array}{c} \begin{array}{cc} r^{2}\pi & 0\leq h\left(k\right)<H_{1} \end{array}\\ \begin{array}{cc} {\left(r+a\left(\cos\left(2\pi f_{r}\left(h\left(k\right)-H_{1}\right)\right)-1\right)\right)}^{2}\pi & H_{1}\leq h(k)\leq H_{max} \end{array} \end{array}$$

The liquid level *h* is chosen as the controlled variable, while the inflow rate *q_in_* is considered the manipulated input. The outflow valve opening *x_valve_* is included as a non-manipulated input and is not subject to direct control. A delay of 1000 sampling instants is applied to the inflow rate, and a delay of 500 sampling instants is applied to the valve opening. The system is simulated using a sampling period of 0.1 s. All system parameter values are summarized in Table 3, and the input ranges are presented in Table 4.

### 2.2. Data Generation and Model Creation

#### 2.2.1. Data Generation

Data for model identification and subsequent MPC evaluation were generated through open-loop simulations of the two benchmark systems described in Section 2.1.1 and Section 2.1.2. Open-loop operation was selected to ensure sufficient excitation of the system dynamics over the relevant operating ranges, without the influence of feedback control. All input signals were generated as multi-level pseudo-random binary sequences with randomly varying dwell times between amplitude changes. This type of excitation is commonly used in system identification [36] due to its ability to sufficiently excite nonlinear dynamics. To obtain data at sampling rates suitable for MPC implementation, the simulated trajectories were downsampled after generation. In the case of the CSTR, every 25th data point was retained, resulting in an effective sampling period of 2.5 s. For the single tank system, every 100th data point was retained, corresponding to an effective sampling period of 10 s. The downsampling was performed to ensure that sufficient computational time would be available between consecutive samples for solving the MPC optimization problem. The different sampling periods reflect the distinct dynamic characteristics of the two systems. After downsampling, each dataset consisted of 12,500 data points. For both systems, the first 10,000 samples were used for training and validation, while the remaining 2500 samples were reserved exclusively for testing and were not used during model training. All input and output variables were normalized to the range [−1,1] prior to model training.

#### 2.2.2. Long Short-Term Memory Networks

**Figure 2:**
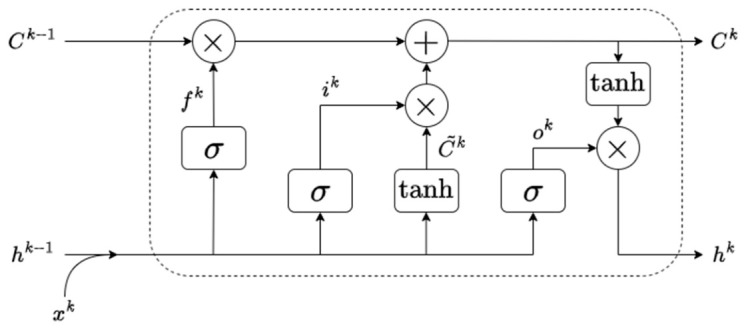
Schematic representation of an LSTM.

LSTM networks [15] are a special type of RNN which were developed to address the vanishing and exploding gradient problem. Unlike a simple RNN, an LSTM network can capture both long-term and short-term temporal dependencies, which has made it one of the most widely used tools for modelling time-dependent sequential data. In addition to the hidden state *h*, LSTMs also include a cell state *C*, which greatly improves the network’s ability to retain long-term information. Furthermore, LSTM neurons introduce gating mechanisms that regulate the flow of data and help limit gradient magnitude during training. In particular, an LSTM neuron contains three gates: the input gate, forget gate, and output gate. The input gate *i* determines how the new input vector ***x*** influences the hidden and cell states, the forget gate *f* determines what proportion of the previous cell state is retained, and the output gate *o* computes the value of the new hidden state [3]. Figure 2 depicts a schematic representation of an LSTM neuron, and the corresponding equations are given in (9)–(14). The symbols ***W_i_***, ***W_h_***, and *b* represent the input weights, recurrent (hidden state) weights, and biases for each gate, respectively. The current time step is represented by *k*. It should be noted that the use of LSTM networks in this work is motivated by the advantages they offer compared to FFNNs and simple RNNs mentioned in the Introduction. However, the proposed method is not restricted to this architecture and can be applied to any model class capable of faithfully representing the system dynamics.

(9) $$i^{k}=\sigma \left(W_{i}^{i}x^{k}+W_{h}^{i}h^{k-1}+b^{i}\right)$$

(10) $$f^{k}=\sigma \left(W_{i}^{f}x^{k}+W_{h}^{f}h^{k-1}+b^{f}\right)$$

(11) $${\tilde{C}}^{k}=\tanh\left(W_{i}^{c}x^{k}+W_{h}^{c}h^{k-1}+b^{c}\right)$$

(12) $$C^{k}=f^{k}C^{k-1}+i^{k}{\tilde{C}}^{k}$$

(13) $$o^{k}=\sigma \left(W_{i}^{o}x^{k}+W_{h}^{o}h^{k-1}+b^{o}\right)$$

(14) $$h^{k}=o^{k}\tanh\left(C^{k}\right)$$

#### 2.2.3. Model Training and Testing

Multiple LSTM network configurations with varying numbers of hidden layers and neurons per hidden layer were considered. Additionally, two variants were implemented for each configuration, motivated by the experiments presented in [37]: one with an output feedback loop and one without it. In the variant with feedback, the output from the previous time step was fed back as an additional input to the network. The output layer consisted of a single fully connected neuron with a linear activation function. All models were trained using the generated datasets described in Section 2.2.1. The mean squared error (MSE) was used as the training loss function, while the coefficient of determination (*R*^2^) was employed as the validation metric to assess predictive performance. For each system, 85% of the available training data was used for parameter optimization, and the remaining 15% was reserved for validation. A comprehensive hyperparameter optimization was not performed, as the primary objective of this work is not to obtain the best possible predictive model but rather to evaluate the proposed algorithm. Consequently, the selected architectures are intended to provide sufficiently accurate models while maintaining a reasonable training complexity. After training, all candidate models were evaluated using the previously unseen test dataset described in Section 2.2.1. The model achieving the highest *R*^2^ score on the test data was selected for subsequent use in the MPC framework.

### 2.3. Prediction Horizon Estimation Algorithm

Selecting an appropriate prediction horizon is a crucial step in MPC design, especially for systems with significant transport delay. As stated earlier, the prediction horizon must be sufficiently long to capture the effect of manipulated inputs on the controlled output. This means that if the chosen prediction horizon is shorter than the input–output delay, the controller is unable to anticipate the system response, leading to degraded closed-loop performance. Cross-correlation is a well-established method for analysing the temporal relationship between two signals. Formally, cross-correlation between two discrete signals *x*(*k*) and *y*(*k*) is a function of the lag *n* that measures the similarity between *x*(*k*) and a shifted version of *y*(*k*). The mathematical formula is given by (15) [38], where *n* represents the lag expressed in samples, *R_xy_*(*n*) the resulting cross-correlation function, and *N* the length of the signals. By definition, *n* ranges from −(*N* − 1) to (*N* − 1); however, it should be noted that only the lags greater than or equal to 0 are considered in this algorithm, as negative lags do not correspond to a causal system.

(15) $$R_{xy}\left(n\right)=\sum_{k=max\left\{0,n\right\}}^{min\left\{N-1,\;N-1+n\right\}}x\left(k\right)y(k-n)$$

**Figure 3:**
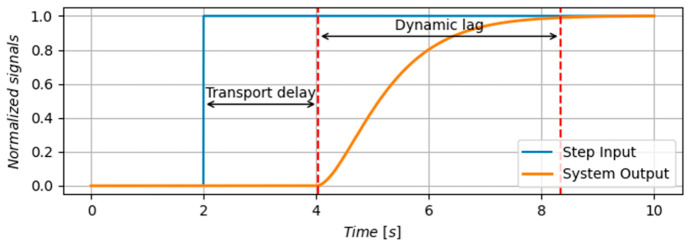
Example of the total delay of a system’s output signal with respect to the input signal.

In the context of dynamic systems, cross-correlation has been used for determining the delay between input and output signals [39,40]. Since the total delay of the output signal with respect to the input signal is the result of both transport delay and the dynamic lag, the latter arising from the system dynamics, the estimated delay covers a time window where the effects of the input signal on the output signal are clearly observable. An example of this is illustrated in Figure 3. Following [41], the prediction horizon should be long enough to capture the significant dynamics of the process. Selecting the prediction horizon based on the combined effect of the transport delay and the dynamic lag ensures that the dominant transient behaviour is captured within the optimization window. Therefore, this work proposes a new method for estimating a suitable prediction horizon for MPC by analysing the cross-correlation function of the manipulated input and the controlled variable of the controlled system. The proposed algorithm operates on simulated input and output data generated using the previously trained LSTM model of the system. The simulations are based on exciting the LSTM model by changing the manipulated input while keeping the non-manipulated inputs at a fixed, constant value, and analysing the temporal relationship between the input signal and the model’s response. Specifically, variations of a step signal with different initial values and step amplitudes are used. Before applying the step change to the manipulated input signal, the model is first warmed up by running simulations with the initial value of the step signal until steady state is reached. Upon reaching steady state, the step change is applied, and the simulation is run further until steady state is reached again. Depending on the applied input signals, the model takes a different amount of time to reach steady state, which affects the lengths of the signals. The lengths of the signals in turn affect the resulting cross-correlation function. However, given that this approach ensures the dynamics of the model are captured, the position of the peak of the cross-correlation function should not be affected by different lengths of the signals. During simulations, all inputs represent normalized values of the input variables in order to suit the trained LSTM network’s input range of [−1, 1]. The proposed initial values and step changes of the manipulated input as well as the values of the non-manipulated inputs are selected to sufficiently excite the LSTM model and enable the observation of representative responses over the majority of its dynamic range. The values are presented in Table 5. Simulations are run for each combination of the manipulated input initial values, step change values and non-manipulated input values, and the corresponding output trajectories of the LSTM model are saved. For the benchmark systems used in this work, with one manipulated and one non-manipulated input, a total of 280 input–output signal pairs are obtained.

**Figure 4:**
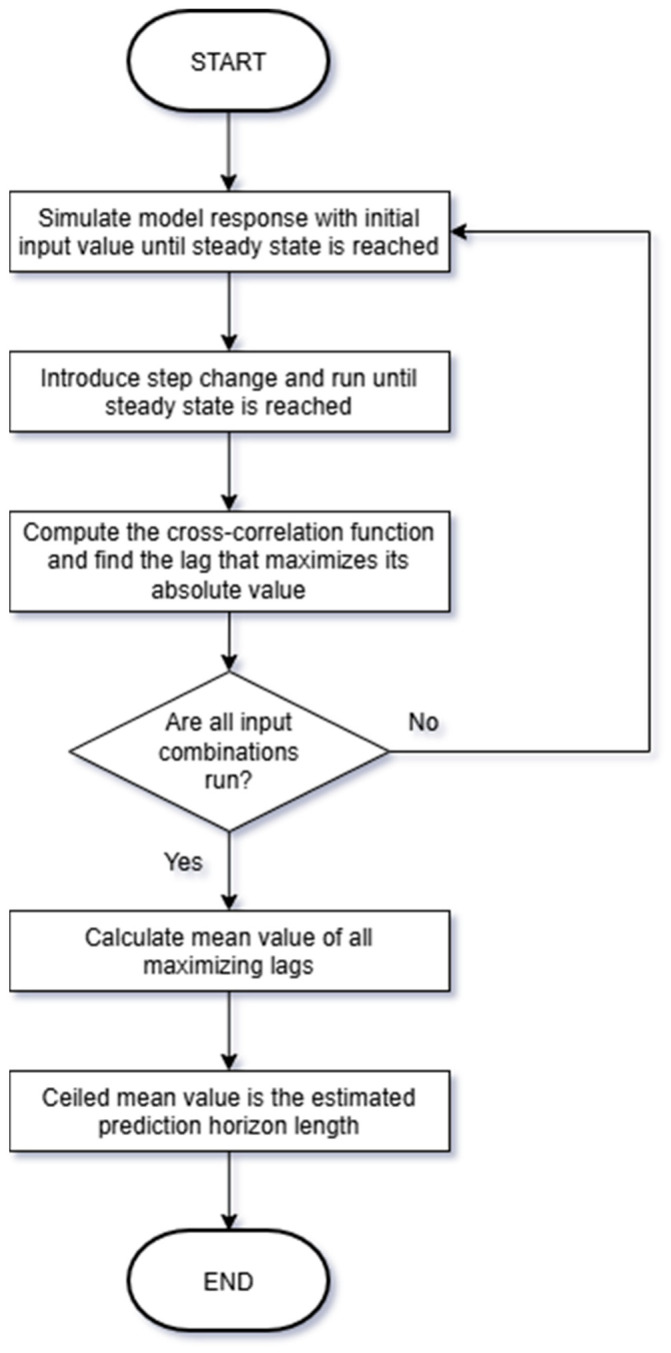
Flowchart of the proposed algorithm.

Prior to cross-correlation analysis, both input and output signals are zero-centred to remove any constant offsets. For each input–output pair, the cross-correlation function is first computed. Its absolute value is then taken to account for both direct and indirect proportionality between signals. Next, the lag corresponding to the maximum value of the absolute cross-correlation is determined. Once all lags corresponding to the maximum value are obtained, their mean value is computed. The prediction horizon is finally selected as the ceiling of this mean value expressed in discrete time steps. A flowchart of the proposed algorithm is presented in Figure 4.

### 2.4. Evaluation Process

The proposed algorithm is evaluated by using the estimated prediction horizons in an MPC framework applied to the two benchmark systems described in Section 2.1.1 and Section 2.1.2. It should be noted that the MPC framework employed in this work is chosen purely for demonstration purposes, serving as a platform on which the effectiveness of the proposed prediction horizon estimation algorithm can be evaluated. No claim is made regarding the superiority of this formulation over other existing MPC methods, as such a comparison falls outside the scope of this work. The used MPC formulation is given by (16)–(20).

(16) $$\underset{\left\{∆u\left(k+1\right),\ldots ,∆u\left(k+N_{c}\right)\right\}}{\min}\sum_{i=1}^{N_{p}}{\left(x^{*}(k+i)-\hat{x}(k+i)\right)}^{2}+P$$

(17) s.t. u(n+1)=u(n)+∆u(n+1)

(18) $$\hat{x}\left(n+1\right)=f_{LSTM}\left(\hat{x}\left(n\right),\;u(n)\right)$$

(19) $$\left|∆u(n)\right|\leq ∆u_{max}$$

(20) $$P=\{\begin{array}{cc} 0 & u_{min}\leq u\left(k+n\right)\leq u_{max},\;n\in \left\{1,\ldots ,N_{p}\right\}\\ C_{max} & else \end{array}$$

The objective is to minimize the cost function over the prediction horizon *N_p_*, with respect to the system dynamics and actuator constraints, starting from the current time step *k*. A genetic algorithm (GA) is employed to solve the nonlinear MPC optimization problem. The GA is chosen as the optimization method because it allows the MPC formulation to handle nonlinear dynamics and nonconvex objective functions without requiring gradient information [42,43,44]. The decision variables are defined as arrays of increments of the manipulated input over the control horizon *N_c_* (Equation (21)) rather than absolute input values. This formulation implicitly limits the rate of change of the control action without introducing an additional tuning parameter for rate penalization. The first element is the increment at time step *k* + 1, the second at time step *k* + 2, and so on until the final element which is the increment at step *k* + *N_c_*.

(21) $$∆u=\left[∆u\left(k+1\right),∆u\left(k+2\right),\ldots ,∆u(k+N_{c})\right]$$

The input increments are constrained to ±10% of the full range of the manipulated input, ±8000.0 cal·s^−1^ for the CSTR and ±4.0 L·s^−1^ for the single tank system. The cost function Equation (15) is defined as the sum of squared errors between the predicted output $\hat{x}$ and the reference trajectory x* over the prediction horizon. To enforce actuator constraints, a hard penalty is applied if the manipulated input exceeds its admissible range at any point over the prediction horizon. The penalty, represented by *P* in Equations (15) and (19), is implemented by adding the maximum cost value of the current generation *C_max_* to the individual’s cost. This way, the penalized individuals become less feasible than the least feasible solution which effectively lowers their probability of influencing individuals of the next generation. In this study, the control horizon is set to the same value as the prediction horizon in order to avoid including additional adjustable parameters. The GA parameters are selected empirically through preliminary experimentation and are summarized in Table 6. In all evaluation experiments, MPC is executed on the corresponding first-principles models, which served as the reference representations of the true system dynamics. The LSTM models are used exclusively for multi-step ahead predictions during optimization within the MPC algorithm, thereby introducing a realistic model–plant mismatch. For each benchmark system, two control scenarios are considered. In the first scenario, the system is regulated to a constant setpoint, while a sudden change is introduced in a non-manipulated input, representing an external disturbance. In the second scenario, a change in the setpoint is applied, while the non-manipulated input is kept at a constant value. In both cases, the control objective is to regulate the controlled variable to the desired setpoint, while rejecting the effects of the non-manipulated inputs. Each scenario is simulated for a duration of 300 sampling instants. Quantitative performance is evaluated using the following metrics: integral of time-weighted absolute error (ITAE), settling time, and overshoot.

### 2.5. Evaluation with Alternative Prediction Horizon Choices

To assess the effectiveness of the proposed prediction horizon estimation method, the MPC performance results obtained using the estimated prediction horizons are compared with multiple alternative prediction horizon selections. Specifically, 3 shorter and 3 longer prediction horizons are considered for each benchmark system. For each prediction horizon, closed-loop MPC simulations are performed under the same test scenarios as described in Section 2.4. The same performance metrics as in the previous section are used, with the addition of mean computation time. All simulations were performed on a PC equipped with an 11th Gen Intel Core i5-11400H CPU (6 cores, 2.69 GHz base frequency; Intel Corporation, Santa Clara, CA, USA) and 32 GB of RAM.

## 3. Results

This section presents the results obtained using the proposed prediction horizon estimation algorithm. First, the predictive accuracy of the trained LSTM models is briefly assessed to verify their suitability for MPC prediction. The estimated prediction horizons are then reported, followed by an evaluation of closed-loop MPC performance using these horizons. Finally, the results are compared with alternative horizon choices to highlight the effectiveness of the proposed approach.

### 3.1. LSTM Model Prediction Performance

**Figure 5:**
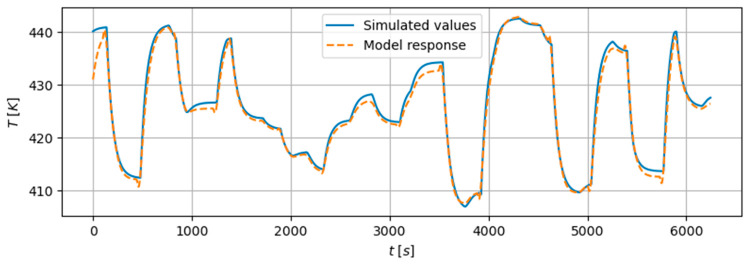
Comparison of the CSTR output predicted by the LSTM model and the simulated system output for the same input sequence.

**Figure 6:**
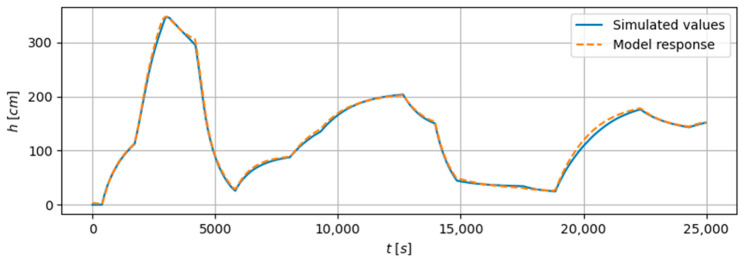
Comparison of the single tank system output predicted by the LSTM model and the simulated system output for the same input sequence.

The predictive performance of the trained LSTM models was evaluated using the test datasets described in Section 2.2. The configurations of the selected models are reported in Table 7, while the corresponding performance metrics for both benchmark systems are summarized in Table 8. The formulas for the performance metrics are given in Equations (22)–(26), where *y* represents the measured signal value, $\hat{y}$ the predicted value, *µ* the mean value of the measured signal, and *N* the length of the signal. The LSTM models for both systems exhibit similar overall architectures; however, the CSTR model showed better performance without a feedback loop, whereas the single tank system performed better with feedback. For both the CSTR and the single tank system, the models achieve high *R*^2^ values, as well as low error-based metrics, on previously unseen test data, indicating that the system dynamics are captured with sufficient accuracy for use in MPC prediction. Figure 5 shows a comparison between the predicted and measured output trajectories for the CSTR, while Figure 6 presents the corresponding results for the single tank system. In both cases, the LSTM models accurately reproduce the behaviour of the systems, including the delayed responses to input changes. These results confirm that the selected models are suitable for multi-step prediction within the MPC framework.

(22) $$MSE=\sum_{i=1}^{N}{\left(y_{i}-\hat{y_{i}}\right)}^{2}$$

(23) $$NMSE=\frac{MSE}{\sum_{i=1}^{N}{\left(y_{i}-\mu \right)}^{2}}$$

(24) $$RMSE=\sqrt{MSE}$$

(25) $$NRMSE=\frac{RMSE}{\underset{i\in \left[1,N\right]}{\max}\left(y_{i}\right)-\underset{i\in \left[1,N\right]}{\min}\left(y_{i}\right)}$$

(26) $$R^{2}=1-\frac{\sum_{i=1}^{N}{\left(y_{i}-\hat{y_{i}}\right)}^{2}}{\sum_{i=1}^{N}{\left(y_{i}-\mu \right)}^{2}}$$

### 3.2. Estimated Prediction Horizons

**Figure 7:**
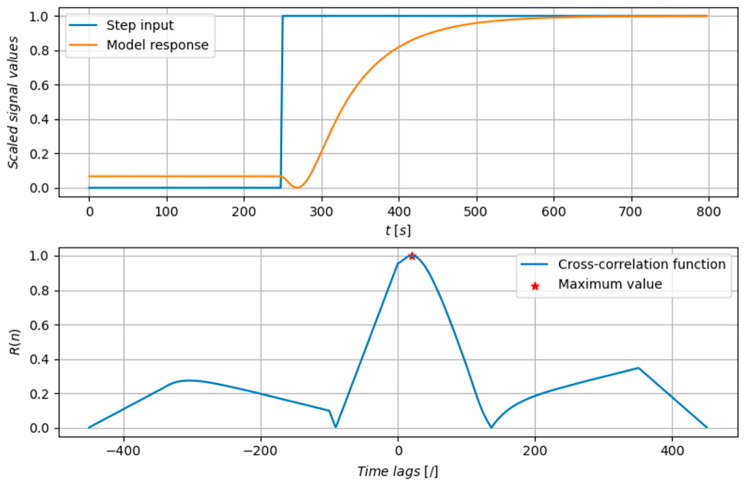
**Top**: Scaled values of the step signal applied to the manipulated input of the LSTM model and the corresponding model response. **Bottom**: Scaled cross-correlation function of the two signals and its maximum value.

The proposed cross-correlation-based algorithm was applied to the trained LSTM models to estimate suitable prediction horizons for both benchmark systems. Using the step–response simulations described in Section 2.3, a representative prediction horizon was obtained for each system by averaging the lags which maximize the cross-correlation function across all simulations, with the final horizon selected as the ceiling of this average expressed in discrete time steps. An example of the scaled step signal and the model’s response, as well as the corresponding normalized cross-correlation function is presented in Figure 7. The resulting prediction horizons are summarized in Table 9 and are used in the subsequent closed-loop MPC performance evaluation. For the first benchmark system, the obtained results exhibit relatively low variability, as suggested by the small standard deviation. In contrast, the second benchmark system exhibits a considerably larger standard deviation. This increased variability can be attributed to the stronger nonlinear behaviour of the system, which causes its dynamics to vary across different states. As a result, the system’s response is less uniform over the operating range, leading to greater dispersion in the obtained results.

To further investigate the influence of prediction model accuracy, the results obtained using models with lower predictive performance than the selected models are presented in Table 10. The results indicate that the prediction horizons estimated using these models are nearly identical to those obtained with the best-performing model, suggesting that the proposed approach does not rely on a perfectly accurate prediction model. The only noticeable deviation is observed for the second benchmark system, where a slightly larger prediction horizon is estimated. However, the difference remains small, and the estimated value is still in close agreement with that obtained using the best-performing model.

### 3.3. Closed-Loop MPC Performance Using the Estimated Prediction Horizons

**Figure 8:**
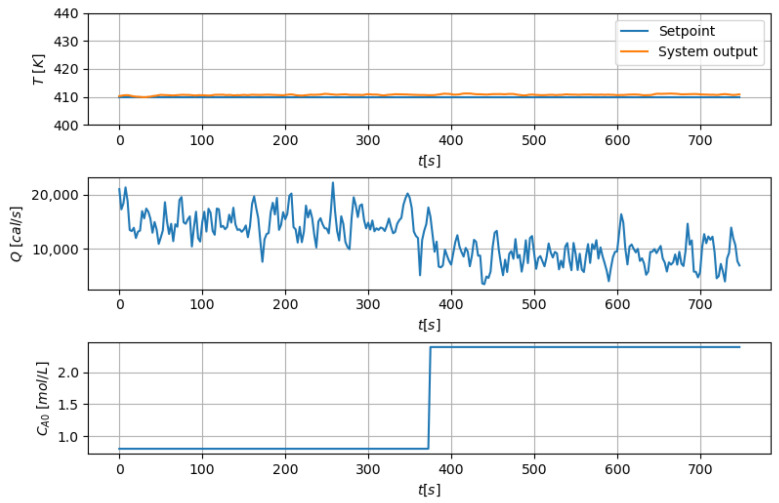
Closed-loop MPC response of the CSTR system for the disturbance rejection scenario using the estimated prediction horizon.

**Figure 9:**
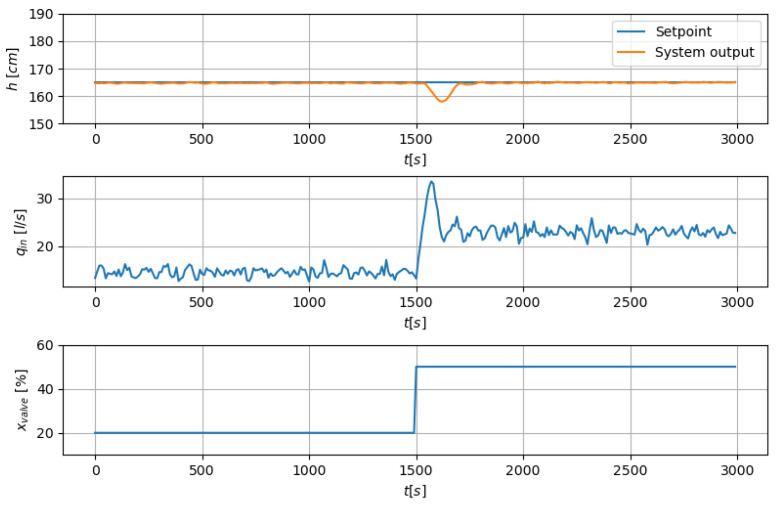
Closed-loop MPC response of the single tank system for the disturbance rejection scenario using the estimated prediction horizon.

**Figure 10:**
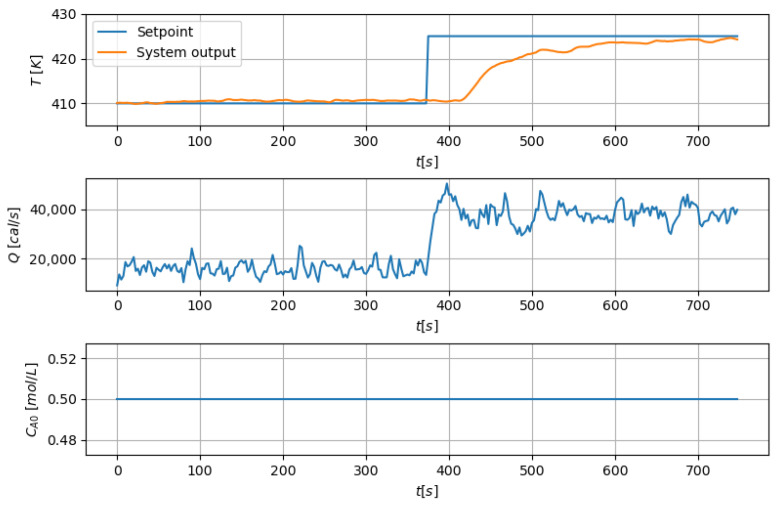
Closed-loop MPC response of the CSTR system for the setpoint tracking scenario using the estimated prediction horizon.

**Figure 11:**
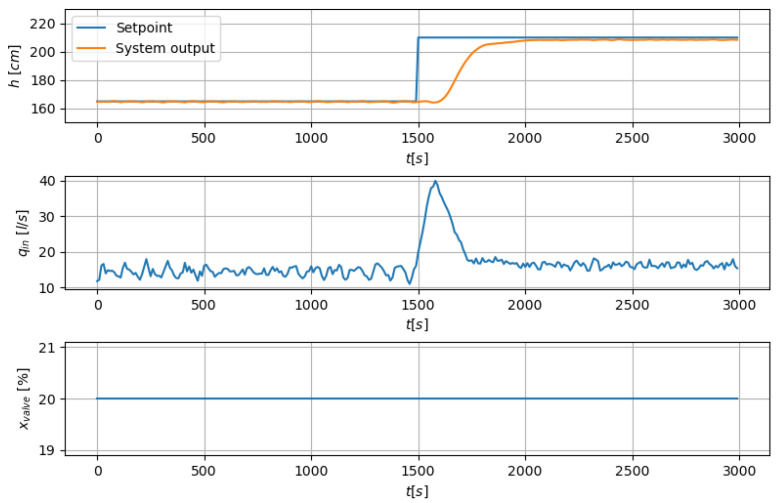
Closed-loop MPC response of the single tank system for the setpoint tracking scenario using the estimated prediction horizon.

Closed-loop MPC simulations were conducted using the prediction horizons obtained in Section 3.2. Figure 8 and Figure 9 present the inputs and closed-loop responses of the controlled variables for the CSTR and the single tank system, respectively, in the first evaluation scenario, in which the setpoint was held constant while the non-manipulated input was subjected to a change. Figure 10 and Figure 11 show the results for the two benchmark systems in the second evaluation scenario, where the non-manipulated input was held constant and the setpoint was varied. The metrics used to evaluate MPC performance are the ITAE, settling time, and overshoot. The obtained results are presented in Table 11 for the first test scenario, and in Table 12 for the second test scenario.

In the disturbance rejection scenario, both benchmark systems exhibit satisfactory closed-loop performance, with the MPC controller successfully rejecting the effects of the change in the non-manipulated input and driving the controlled variable to steady state. For the CSTR, the effect of the disturbance is minimal, which is also reflected in the settling time of 0. The controlled variable never crossed the ±2% boundary of the setpoint, however, based on the drop in the mean value of the manipulated input signal, it is clear that the MPC controller responded accordingly to the disturbance. In the case of the single tank, similar behaviour was observed, with the difference being a more pronounced effect of the disturbance on the controlled variable. This difference is mainly due to the differences in system dynamics between the two systems. In the setpoint tracking scenario, both the CSTR and the single tank system achieve accurate tracking of the setpoint change and reach steady state. Furthermore, the controlled variables of both systems reach their respective steady states without producing an overshoot.

The overall responses remain stable and demonstrate the ability of the estimated prediction horizons to immediately react on both disturbances and setpoint changes.

### 3.4. Comparison with Alternative Prediction Horizon Choices

Closed-loop MPC simulations were conducted using the alternative prediction horizons as stated in Section 2.5. The resulting performance metrics for all tested prediction horizons are summarized in Table 13 for both systems in the first test scenario and in Table 14 for the second test scenario. When shorter prediction horizons were used, the two systems exhibited different behaviour. For the CSTR, the MPC controller could not keep the controlled variable at the setpoint in either of the test scenarios. The results show that the ITAE decreased with each increase in the prediction horizon up to the value estimated in Section 3.2; however, none were enough to enable successful control. The single tank system exhibited similar behaviour for the shortest prediction horizon of nine time steps, where neither the effect of the disturbance was rejected nor was the change in the setpoint tracked. The remaining two shorter prediction horizons, 12 and 15 time steps, were able to successfully reject the effects of the disturbance and lead the controlled variable to the new setpoint. In these cases, however, the setpoint was reached with delay compared to the prediction horizon estimated in Section 3.2, which is reflected in both the ITAE and settling time values.

Increasing the prediction horizon beyond the estimated value also led to different effects across the two systems. For the CSTR, longer prediction horizons resulted in reduced settling times and lower ITAE values, although at the cost of slight overshoots, particularly in the second test scenario. Additionally, the ITAE values started rising with the increase in the prediction horizon beyond 23 time steps. For the single tank system, the ITAE values remained largely unchanged, exhibiting slight increases, while the settling time was reduced by a few time steps in both evaluation scenarios. As expected, the mean computation time increased with longer prediction horizons for both systems; however, all evaluated prediction horizons remained computationally feasible with respect to the available sampling period.

The increase in ITAE values for both systems when using longer prediction horizons can be attributed to the parameters of the GA, such as insufficient number of generations or individuals in a population, as well as to the model accuracy. Because the control horizon was set equal to the prediction horizon, increasing the prediction horizon also increased the dimensionality of the optimization problem, potentially degrading the performance of the GA. To address this issue, additional simulations were performed for the three longest prediction horizons for both benchmark systems where the control horizon was set to the value determined by the proposed method and the number of generations and individuals in a population were increased by 50%. Since the resulting ITAE values showed no significant improvement, it was concluded that the degradation in performance was primarily caused by model inaccuracies rather than insufficient GA tuning. Furthermore, the increased parameters of the GA led to longer computation times and rendered the MPC algorithm infeasible for the CSTR with respect to its sampling period.

Given that the transport delays for these systems are known and measure 16 and 10 time steps for the CSTR and single tank system, respectively, a further conclusion can be drawn. The prediction horizons of 11 and 14 time steps for the CSTR and nine time steps for the single tank system are lower than the transport delays of their respective manipulated inputs. Therefore, it is not surprising that the MPC controller failed to control the systems using these prediction horizons, as they are insufficient to capture the effects of the manipulated input over the prediction horizon. For the CSTR, even the prediction horizon of 17 time steps, which effectively predicts one time step influenced by the manipulated input, was insufficient to establish successful control. This result confirms that prediction horizons should cover multiple time steps affected by the manipulated input.

Overall, the quantitative results reported in Table 13 and Table 14 indicate that the prediction horizons obtained using the proposed algorithm are sufficiently long to ensure effective closed-loop control despite the presence of transport delay. The estimated prediction horizons consistently achieve performance comparable to that of longer prediction horizons, while avoiding the performance degradation associated with shorter prediction horizon choices.

## 4. Discussion

The results presented in this paper demonstrate that the proposed cross-correlation-based prediction horizon estimation algorithm provides a practical and reliable means of selecting an appropriate prediction horizon for MPC control of nonlinear systems with transport delay. The key novelty of the method lies in training a data-driven model on data collected during normal process operation, which is then used to simulate the system’s response to step-based inputs, eliminating the need to apply potentially disruptive excitation signals directly to the system, as would be required by conventional identification methods. This is particularly significant in real industrial environments, where such excitation is often both impractical and unsafe. Consequently, the presented method can determine a suitable prediction horizon beforehand, which is another important advantage. Unlike the approach in [37], where the prediction horizon is chosen and refined experimentally, the proposed method yields a clear and well-defined prediction horizon without the need for additional adjustment procedures. Furthermore, in applications where the system dynamics vary over time or where the process operates in multiple regimes with distinct dynamic characteristics, adjustment of the prediction horizon may become necessary. In such cases, several of the methods discussed in the Introduction could be employed. Nevertheless, the proposed method still provides a suitable initial prediction horizon, enabling safe and immediate deployment while reducing the need for extensive preliminary tuning.

The obtained results clearly indicate that prediction horizons shorter than the estimated value are often insufficient to capture the dominant process dynamics, particularly in the presence of transport delay. In both benchmark systems, the use of overly short horizons led to slow convergence or complete failure to reach the desired setpoint, with this effect being especially pronounced in the CSTR system. These observations confirm that the prediction horizon must exceed the transport delay associated with the manipulated input to enable the MPC to formulate an effective control strategy. At the same time, increasing the prediction horizon beyond the estimated value yielded diminishing performance improvements. For the CSTR system, longer horizons reduced settling times and ITAE values but introduced overshoot, while for the single tank system, the impact on ITAE was marginal, and only limited improvements in settling time were observed. These findings suggest that excessively long prediction horizons may not yield meaningful performance improvements, while inevitably increasing computational cost. However, it is important to emphasize that the proposed method does not provide a globally optimal prediction horizon, but rather a feasible and suitable one that ensures the optimization window is long enough to capture the dynamic response of the controlled variable to changes in the manipulated input, thereby yielding satisfactory control performance. While longer horizons may in some cases produce improved ITAE values, the estimated horizon is intended to provide a reliable and practically viable starting point, without any requirement for system excitation or arbitrary initialization. Although the method relies on the availability of a sufficiently accurate prediction model, the observed robustness to model–plant mismatch across both benchmark systems suggests that the approach can be effectively applied in practical MPC settings. Furthermore, gradual changes in the system dynamics due to aging or wear or changes in working conditions do not represent a limitation of the proposed approach. In such cases, the prediction model can be retrained using newly acquired process data to accurately capture the current system behaviour. The proposed prediction horizon estimation procedure can then be repeated to determine a prediction horizon appropriate for the updated dynamics.

Overall, the results support the proposed prediction horizon estimation algorithm as an effective and practical approach for determining a suitable prediction horizon in MPC design for nonlinear systems with transport delay. However, several limitations of the present study should be acknowledged. First, the proposed method has been evaluated only on benchmark systems, and its performance on real industrial processes remains to be validated experimentally. Second, the current analysis was performed on systems with one manipulated input and one controlled variable, which are very common in the process industry. Future work will investigate the extension of this method to systems with multiple manipulated inputs and controlled variables. Thirdly, this method relies heavily on the availability of a model of the controlled process. If a process is controlled manually or by a conventional controller, the gathered data may not contain much information about the dynamic behaviour of the process, which makes training a model difficult. Finally, because the prediction horizon determination procedure operates on data generated by a trained LSTM model, measurement noise affects the method indirectly through the model training stage rather than the horizon estimation process itself. Therefore, the impact of noisy or uncertain training data on the resulting prediction horizon should be examined in future work.

## Figures and Tables

**Table 1:** List of the parameters for the CSTR and their values.

Parameter Values	
$T_{0}=400\;K$	τ=60 s
$k_{A}=5000\;1/s$	V=100 L
$k_{B}=10^{6}\;1/s$	$E_{A}=1\cdot 10^{4}\;cal/mol$
R=1.987 cal/(mol·K)	$E_{A}=1.5\cdot 10^{4}\;cal/mol$
ρ=1 kg/L	∆H=−5000 cal/mol
$C_{p}=1000\;cal/\left(kg\cdot K\right)$	

**Table 2:** Input ranges for the CSTR.

Heating Rate	Inlet Concentration
Q∈[386.0, 80,386.0] cal/s	$C_{A0}\in \left[0.0,\;3.0\right]\;mol/L$

**Table 3:** List of the parameters for the single tank system and their values.

Parameter Values	
$C_{d}=0.6$	a=20 cm
$D_{out}=11\;cm$	$H_{1}=50\;cm$
$g=9.81\;m/s^{2}$	$H_{max}=346\;cm$
r=199.5 cm	

**Table 4:** Input ranges for the single tank system.

Inflow Rate	Outflow Valve Opening
$q_{in}\in \left[0.0,\;40.0\right]\;L/s$	$x_{valve}\in \left[0.0,\;100.0\right]\%$

**Table 5:** Input data for the manipulated and non-manipulated inputs during simulations.

Parameter	Values
Manipulated input initial values	{0.0, ±0.25, ±0.65}
Step changes	{±0.05, ±0.1, ±0.15, ±0.2}
Non-manipulated inputs values	{0.0, ±0.3, ±0.6, ±0.9}

**Table 6:** The values of the parameters used for the GA.

Parameter	Value
Population size	50
Generations	10
Crossover fraction	80%
Elitism fraction	10%
Mutation fraction	10%
Crossover method	Blend crossover (BLX-α)
α	0.2

**Table 7:** Configurations of the selected LSTM models used for the MPC simulations for each benchmark system.

Hyperparameter	CSTR	Single Tank System
No. of hidden layers	3	3
No. of units per hidden layer	8	12
No. of inputs	2	3

**Table 8:** Predictive performance metrics of the selected LSTM models evaluated on the test datasets.

Metric	CSTR	Single Tank System
Mean squared error (MSE)	1.408	16.429
Normalized mean squared error (NMSE)	0.0141	0.0026
Root mean squared error (RMSE)	1.186	4.053
Normalized root mean squared error (NRMSE)	0.033	0.0117
Coefficient of determination ($R^{2}$)	0.986	0.997

**Table 9:** Mean values and standard deviations of the lags that maximize the cross-correlation function and the corresponding estimated prediction horizons for the two benchmark systems.

System	Mean Value and Standard Deviation of the Lags That Maximize the Cross-Correlation Function	Estimated Prediction Horizon
CSTR	19.32 ± 1.16	20
Single tank system	17.50 ± 6.26	18

**Table 10:** Coefficients of determination and mean values and standard deviations of the lags that maximize the cross-correlation function for the two benchmark systems using prediction models with lower predictive performance than the selected models.

System	R^2^	Mean Value and Standard Deviation of the Lags That Maximize the Cross-Correlation Function
CSTR	0.935	19.92±2.17
	0.959	19.11±1.26
	0.962	19.56±0.63
Single tank system	0.835	19.61±4.12
	0.903	19.89±6.33
	0.981	18.56±6.22

**Table 11:** Closed-loop MPC performance metrics for the disturbance rejection scenario using the estimated prediction horizons. Entries marked as N/A indicate that the corresponding metric is not applicable, either because no overshoot occurred or steady state was not reached within the simulation interval.

System	ITAE	Settling Time [Time Steps]	Overshoot [%]
CSTR	$9.23\cdot 10^{4}$	0	0.300
Single tank system	$1.76\cdot 10^{5}$	17	0.149

**Table 12:** Closed-loop MPC performance metrics for the setpoint tracking scenario using the estimated prediction horizons. Entries marked as N/A indicate that the corresponding metric is not applicable, either because no overshoot occurred or steady state was not reached within the simulation interval.

System	ITAE	Settling Time [Time Steps]	Overshoot [%]
CSTR	$3.28\cdot 10^{5}$	27	N/A
Single tank system	$2.09\cdot 10^{6}$	37	N/A

**Table 13:** Closed-loop MPC performance metrics for the disturbance rejection scenario obtained using the estimated prediction horizon and multiple shorter and longer prediction horizons. Entries marked as N/A indicate that the corresponding metric is not applicable, either because no overshoot occurred or steady state was not reached within the simulation interval.

System	Prediction Horizon [Time Steps]	ITAE	Settling Time [Time Steps]	Overshoot [%]	Mean Execution Time per Sample [s]
CSTR	11	$4.43\cdot 10^{6}$	N/A	N/A	1.10±0.05
	14	$3.13\cdot 10^{6}$	N/A	N/A	1.28±0.03
	17	$2.05\cdot 10^{6}$	N/A	N/A	1.48±0.04
	20	$9.23\cdot 10^{4}$	0	0.300	1.68±0.04
	23	$1.86\cdot 10^{4}$	0	0.117	1.87±0.05
	26	$9.65\cdot 10^{3}$	0	0.099	2.06±0.07
	29	$1.12\cdot 10^{4}$	0	0.147	2.24±0.06
Single tank system	9	$1.35\cdot 10^{7}$	N/A	N/A	1.41±0.04
	12	$5.88\cdot 10^{5}$	27	N/A	1.70±0.04
	15	$2.95\cdot 10^{5}$	18	N/A	1.99±0.05
	18	$1.76\cdot 10^{5}$	17	0.149	2.25±0.05
	21	$1.96\cdot 10^{5}$	17	0.407	2.51±0.03
	24	$1.95\cdot 10^{5}$	16	0.504	2.85±0.06
	27	$1.89\cdot 10^{5}$	16	0.405	3.15±0.09

**Table 14:** Closed-loop MPC performance metrics for the setpoint tracking scenario obtained using the estimated prediction horizon and multiple shorter and longer prediction horizons. Entries marked as N/A indicate that the corresponding metric is not applicable, either because no overshoot occurred or steady state was not reached within the simulation interval.

System	Prediction Horizon [Time Steps]	ITAE	Settling Time [Time Steps]	Overshoot [%]	Mean Execution Time per Sample [s]
CSTR	11	$2.79\cdot 10^{6}$	N/A	N/A	1.08±0.02
	14	$1.85\cdot 10^{6}$	N/A	N/A	1.26±0.04
	17	$6.96\cdot 10^{5}$	N/A	N/A	1.49±0.06
	20	$3.28\cdot 10^{5}$	27	N/A	1.66±0.02
	23	$1.88\cdot 10^{5}$	26	0.073	1.88±0.04
	26	$1.96\cdot 10^{5}$	25	0.428	2.05±0.05
	29	$2.09\cdot 10^{5}$	25	0.536	2.28±0.09
Single tank system	9	$1.19\cdot 10^{7}$	N/A	N/A	1.40±0.03
	12	$2.79\cdot 10^{6}$	52	N/A	1.70±0.06
	15	$2.22\cdot 10^{6}$	38	N/A	2.01±0.01
	18	$2.09\cdot 10^{6}$	37	N/A	2.28±0.05
	21	$2.09\cdot 10^{6}$	35	N/A	2.57±0.07
	24	$2.11\cdot 10^{6}$	34	N/A	2.78±0.03
	27	$2.13\cdot 10^{6}$	35	N/A	3.13±0.06

## Data Availability

The raw data supporting the conclusions of this article will be made available by the authors on request.

## Abbreviations

The following abbreviations are used in this manuscript:

MPC	Model predictive control
FFNN	Feedforward neural network
RNN	Recurrent neural network
LSTM	Long short-term memory
HVAC	Heating, ventilation and air conditioning
CSTR	Continuous stirred-tank reactor
MSE	Mean squared error
GA	Genetic algorithm
ITAE	Integral of time-weighted absolute error
NMSE	Normalized mean squared error
RMSE	Root mean squared error
NRMSE	Normalized root mean squared error
